# Recurrent spontaneous closure of a full-thickness macular hole following IOL scleral fixation: A case report

**DOI:** 10.1016/j.ajoc.2026.102573

**Published:** 2026-03-24

**Authors:** Marina Ogawa, Yohei Tomita, Yuka Ota, Norimitsu Ban, Hiromitsu Kunimi, Isami Hayashi, Xiaoyan Jiang, Toshihide Kurihara, Hajime Shinoda, Kazuno Negishi

**Affiliations:** a Department of Ophthalmology, Keio University School of Medicine, 35 Shinanomachi, Tokyo, Shinjuku-ku, 160-8582, Japan; b Laboratory of Chorioretinal Biology, Keio University School of Medicine, 35 Shinanomachi, Tokyo, Shinjuku-ku, 160-8582, Japan; c Tokyo Dental College Suidobashi Hospital, 2-9-18 Kanda-Misakicho, Tokyo, Chiyoda-ku, 101-0061, Japan

**Keywords:** Full-thickness macular hole, Spontaneous closure, Vitrectomy, IOL scleral fixation, Epiretinal proliferation

## Abstract

**Purpose:**

To report a rare case of a full-thickness macular hole (FTMH) that underwent spontaneous closure on two separate occasions following vitrectomy and intraocular lens (IOL) scleral fixation.

**Observations:**

A 58-year-old man, who had undergone IOL scleral fixation and vitrectomy for a dislocated IOL, developed an FTMH (minimum diameter: 21 μm) in the left eye approximately one year post-surgery. While awaiting surgical intervention, the macular hole closed spontaneously two months after its discovery. Two years and nine months later, the FTMH recurred (minimum diameter: 180 μm) and closed spontaneously again within four weeks. Optical coherence tomography (OCT) confirmed the presence of epiretinal proliferation (EP) at the macular surface during both occurrences.

**Conclusions and Importance:**

Small-diameter FTMHs that form after vitrectomy, particularly those with associated EP, may have the potential for spontaneous closure. This case indicates that short-term observation may be a reasonable management option for select cases of small post-vitrectomy macular holes before proceeding with further surgery.

## 1 Introduction

The formation of a full-thickness macular hole (FTMH) is a recognized but uncommon complication following pars plana vitrectomy. The pathogenesis of post-vitrectomy FTMH is considered multifactorial. One leading hypothesis involves the development of secondary epiretinal membranes (ERMs). It is thought that tangential traction exerted by these membranes on the foveal surface can lead to structural failure and hole formation.^1^ Another proposed mechanism relates to postoperative inflammation, which can induce cystoid macular edema (CME), creating structural fragility within the fovea and predisposing it to dehiscence.^2^

While surgical intervention remains the standard of care for most FTMHs, spontaneous closure is a rare but documented phenomenon, observed in a small subset of cases 3, 4, 5. The recurrence of an FTMH after an initial spontaneous closure is exceptionally rare. Although a few case reports have documented recurrent spontaneous closure of macular holes, such instances remain highly unusual.^5^ Herein, we report a unique case of a patient who developed an FTMH following vitrectomy with intraocular lens (IOL) scleral fixation and subsequently experienced two distinct episodes of spontaneous closure.

## 2 Case report

**Fig. 1:**
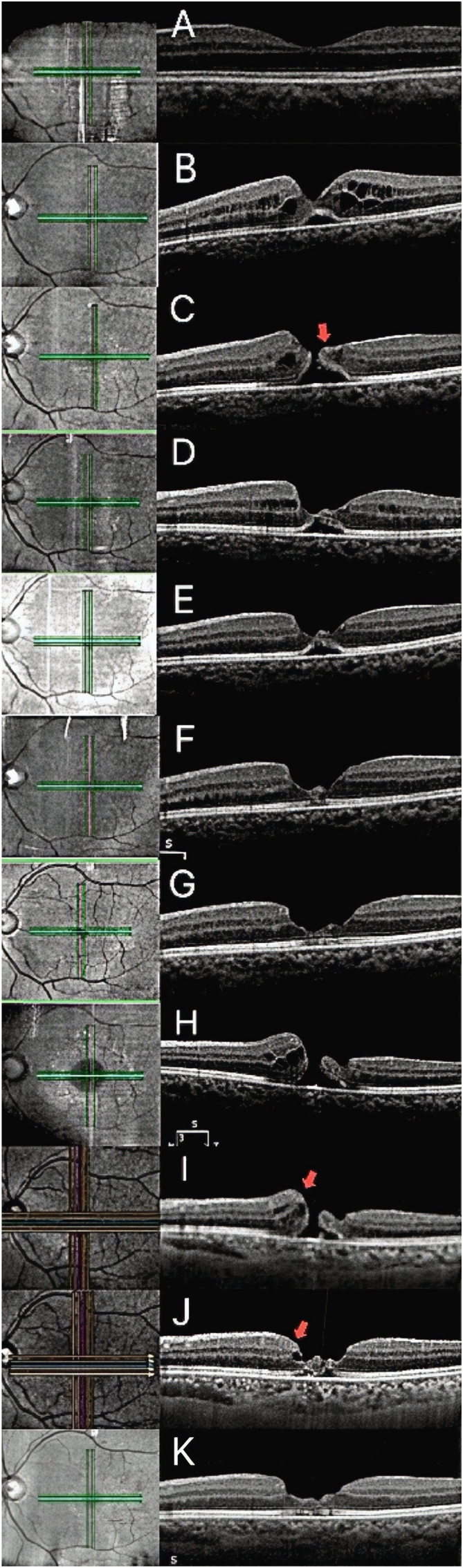
Serial OCT images of the left eye demonstrating the clinical course. **A**: postoperative day 1 after vitrectomy and IOL scleral fixation, **B**: one year after the operation, **C**: the initial FTMH, **D**: spontaneous closure with associated epiretinal proliferation (EP, indicated by red arrowheads), **E**: one month after spontaneous closure, **F**: three months after spontaneous closure, **G**: two years and three months after spontaneous closure, **H, I**: the recurrence of the FTMH, and **J**: the second spontaneous closure, **K**: two months after the second spontaneous closure. (ZEISS CIRRUS 5000 for **A**-**H**, **K;** Canon Xephilio OCT-S1 for **I, J**). (For interpretation of the references to colour in this figure legend, the reader is referred to the Web version of this article.)

A 58-year-old man underwent IOL scleral fixation with concomitant vitrectomy for a dislocated IOL in his left eye. During the surgery, the posterior hyaloid membrane was peeled. His postoperative best-corrected visual acuity (BCVA) was excellent at 20/10. Optical coherence tomography (OCT, ZEISS CIRRUS 5000, Germany, and Canon Xephilio OCT-S1, Japan) images taken the day after surgery and one year after surgery are shown in Fig. 1A and B, respectively.

One year and one month after the surgery, he presented with decreased vision in the left eye. His BCVA had declined to 20/33. OCT confirmed the presence of an FTMH with a minimum diameter of 21 μm (Fig. 1C). The patient was scheduled for vitrectomy, and topical bromfenac sodium hydrate was initiated. However, a follow-up examination two months later revealed that the macular hole had closed spontaneously (Fig. 1D). The patient's BCVA improved to 20/20, and OCT showed resolution of the hole with presence of epiretinal proliferation (EP) over the fovea (Fig. 1D).

**Fig. 2:**
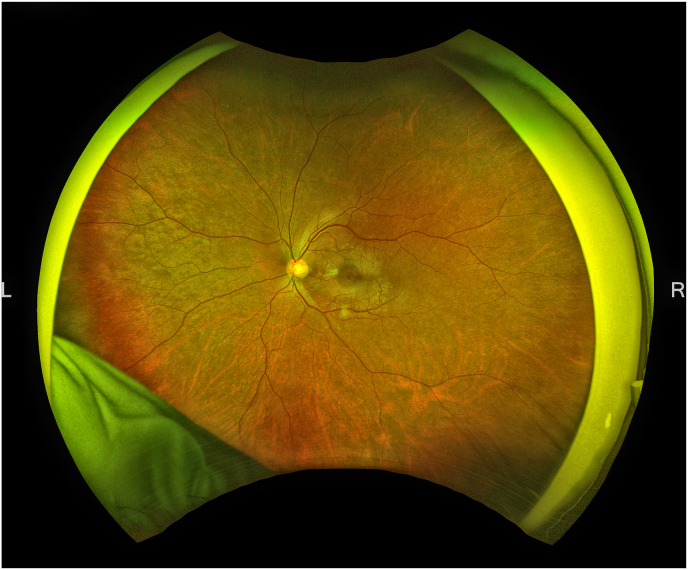
Color fundus photograph of the left eye at the time of macular hole recurrence. (For interpretation of the references to colour in this figure legend, the reader is referred to the Web version of this article.)

The patient remained stable without any recurrence of the FTMH for over two years, as shown in Fig. 1E, F, and G. However, two years and nine months after the initial closure, he again experienced blurred vision in the left eye, with BCVA decreasing to 20/40. OCT imaging confirmed a recurrence of the FTMH, now with a minimum diameter of 180 μm (Fig. 1, Fig. 2). EP was again noted on the macular surface (Fig. 1I). Vitrectomy was planned; however, the hole closed spontaneously within four weeks after recognition of recurrence, prior to the scheduled surgery (Fig. 1J). No topical medications, including eye drops, were administered. His BCVA subsequently improved to 20/28, and the surgical plan was canceled. The subsequent clinical course has been favorable with BCVA increasing to 20/20 (Fig. 1K).

## 3 Discussion

This case report details the highly unusual clinical course of a recurrent, twice-spontaneously closing FTMH following vitrectomy. The spontaneous resolution of an FTMH is a complex event, and its occurrence is largely dictated by a confluence of anatomical and cellular factors at the fovea.

One critical determinant of spontaneous closure is the diameter of the macular hole. Clinical evidence demonstrates that smaller holes are more likely to close without intervention. A large retrospective study by Neubauer et al. demonstrated that the mean FTMH diameter in eyes that underwent spontaneous closure was 98.9 μm, compared to 345.6 μm in those that did not close.^6^ This aligns with other reports suggesting that holes under 250 μm are the most likely candidates for spontaneous resolution.^7^ Our patient's initial hole (21 μm) and recurrent hole (180 μm) both fall within this range, likely serving as a primary factor in the favorable outcomes observed.

Another important factor is the presence of epiretinal proliferation (EP), noted on OCT during both closure events. The precise mechanism by which EP facilitates FTMH closure remains under investigation. Unlike pathogenic ERMs, which are rich in collagen and cause traction, EP is thought to represent reparative tissue primarily composed of proliferating Müller glial cells.^8^^,^^9^ It is hypothesized that these proliferating cells may physically bridge the defect, forming a cellular scaffold that promotes the migration and realignment of photoreceptors, subsequently closing the neurosensory gap.^3^ This glial proliferation may also alter the biomechanical forces at the vitreoretinal interface, potentially counteracting residual tangential traction and thereby creating a more favorable environment for anatomical resolution.^9^^,^^10^ The consistent presence of EP in this case strongly suggests it played a pivotal role in mediating both closure events.

Although scattered case reports have suggested that topical NSAIDs alone can facilitate FTMH closure,^11^ it remains controversial whether these closures were truly drug-induced or merely coincidental with the natural course of spontaneous resolution. Distinguishing between a therapeutic effect and the natural history of the disease is often challenging in retrospective observations. In our case, the initial closure occurred during bromfenac use, which might initially suggest a pharmacological benefit. However, the recurrent hole closed completely without the administration of any topical medications. This distinct clinical course provides compelling evidence that the closures in this eye were primarily driven by intrinsic anatomical factors—specifically the small hole diameter and the presence of EP—rather than pharmacological intervention.

In conclusion, the repeated spontaneous closure in this patient was likely attributable to the combined influence of small hole diameter and the presence of EP. This case highlights that a conservative management approach, involving a period of careful observation, may be a viable strategy for small FTMHs that develop post-vitrectomy, particularly when associated with OCT evidence of EP.

## 4 Conclusion

We have described an exceptional case of an FTMH that recurred and closed spontaneously twice following vitrectomy with IOL scleral fixation. This case underscores that small-diameter macular holes that develop post-vitrectomy, particularly in the presence of epiretinal proliferation, have a significant potential for spontaneous resolution. A period of careful observation may be considered for such cases before recommending surgical intervention.

## CRediT authorship contribution statement

**Marina Ogawa:** Writing – original draft, Data curation, Conceptualization. **Yohei Tomita:** Writing – review & editing, Visualization, Supervision, Conceptualization. **Yuka Ota:** Writing – review & editing, Data curation. **Norimitsu Ban:** Writing – review & editing. **Hiromitsu Kunimi:** Writing – review & editing. **Isami Hayashi:** Writing – review & editing. **Xiaoyan Jiang:** Writing – review & editing. **Toshihide Kurihara:** Writing – review & editing. **Hajime Shinoda:** Writing – review & editing. **Kazuno Negishi:** Writing – review & editing.

## Patient consent

Written consent to publish this case has not been obtained. This report does not contain any personal identifying information.

## Funding

This study was supported by a Grant-in-Aid for Scientific Research (KAKENHI) from the Japan Society for the Promotion of Science (JSPS) (Grant Numbers: JP23K15938, JP25K12855) to Y.T.

## Declaration of competing interest

The authors declare that they have no known competing financial interests or personal relationships that could have appeared to influence the work reported in this paper.
